# Astodrimer sodium antiviral nasal spray for reducing respiratory infections is safe and well tolerated in a randomized controlled trial

**DOI:** 10.1038/s41598-022-14601-3

**Published:** 2022-06-17

**Authors:** Alex Castellarnau, Graham P. Heery, Aynaz Seta, Carolyn A. Luscombe, George R. Kinghorn, Peter Button, Philip McCloud, Jeremy R. A. Paull

**Affiliations:** 1 Starpharma Pty Ltd, 4-6 Southampton Crescent, Abbotsford, VIC 3067 Australia; 2 University of Sheffield, Sheffield, S10 2TN UK; 3 McCloud Consulting Group Pty Ltd, Belrose, NSW 2085 Australia

**Keywords:** SARS-CoV-2, Antivirals

## Abstract

Astodrimer sodium is a dendrimer molecule with antiviral and virucidal activity against SARS-CoV-2 and other respiratory viruses in vitro, and has previously been shown to be safe and well tolerated, and not systemically absorbed, when applied to the vaginal mucosa. To investigate its potential utility as a topical antiviral, astodrimer sodium has been reformulated for application to the nasal mucosa to help reduce viral load before or after exposure to respiratory infection. The current investigation assessed the safety, tolerability and absorption of astodrimer sodium 1% antiviral nasal spray. This was a single-centre, double-blinded, randomized, placebo-controlled, exploratory clinical investigation. Forty healthy volunteers aged 18 to 65 years with no clinically significant nasal cavity examination findings were randomized 3:1 to astodrimer sodium nasal spray (N = 30) or placebo (N = 10) at an Australian clinical trials facility. An initial cohort of participants (N = 12 astodrimer, N = 4 placebo) received a single application (one spray per nostril) to assess any acute effects, followed by a washout period, before self-administering the spray four times daily for 14 days to represent an intensive application schedule. Extent of absorption of astodrimer sodium via the nasal mucosa was also assessed in this cohort. A second cohort of participants (N = 18 astodrimer, N = 6 placebo) self-administered the spray four times daily for 14 days. The primary endpoint was safety, measured by frequency and severity of treatment emergent adverse events (TEAEs), including clinically significant nasal cavity examination findings, in the safety population (all participants randomized who administered any spray). Participants were randomized between 6 January 2021 and 29 March 2021. TEAEs occurred in 8/10 (80%) participants in the placebo arm and 19/30 (63.3%) participants in the astodrimer sodium arm; all were of mild intensity. TEAEs considered potentially related to study product occurred in 5/10 (50%) participants receiving placebo and 10/30 (33.3%) of participants receiving astodrimer sodium. No participants experienced serious AEs, or TEAEs leading to withdrawal from the study. No systemic absorption of astodrimer sodium via the nasal mucosa was detected. Astodrimer sodium nasal spray was well tolerated and is a promising innovation warranting further investigation for nasal administration to potentially reduce infection and spread of community acquired respiratory virus infections.

**Trial Registration:** ACTRN12620001371987, first registered 22-12-2020 (Australia New Zealand Clinical Trials Registry, https://anzctr.org.au/).

## Introduction

Epidemic and pandemic respiratory viral infections have caused significant public health problems worldwide. Recent examples of pandemic viruses include the current severe acute respiratory syndrome coronavirus 2 (SARS-CoV-2), and influenza A virus (H1N1/pdm/09). Each of these pandemics has been associated with the emergence of virus that causes more rapid and severe pneumonia than other strains^1^. Respiratory viral infections are also responsible for seasonal endemics that generally cause relatively mild infection and symptoms, but occasionally are more severe and result in death^2^.

Transmission of respiratory virus occurs when droplets containing virus from an infected individual are expelled a short distance through the air and are either directly delivered to the nasal or oral mucous membranes of a new individual, or transferred by indirect contact with contaminated surfaces^3–5^. The first line of defence against respiratory viral infection includes intrinsic defences such as mucus and innate immune detection and response. A nasal spray to provide a temporary barrier to boost the host’s defence against invasion by a pathogenic virus could limit or prevent viral infection and clinical sequelae.

Astodrimer sodium is a large (3 to 4 nm, ~ 16.5 kDa), negatively charged, highly-branched dendrimer^6^ that has antiviral and virucidal activity against a broad spectrum of viruses^7–11^. Astodrimer sodium has recently demonstrated antiviral and virucidal activity against SARS-CoV-2 in vitro^12^, including the Alpha, Beta, Gamma, Delta^13^ and Omicron Variants of Concern. Astodrimer sodium (1% w/w) in a mucoadhesive nasal spray formulation significantly reduced viral genome copies (> 99.9%) and infectious virus (> 90%) in the lung and trachea compared to saline treatment in K18-hACE2 mice intranasally infected with SARS-CoV-2^14^.

Mechanism of action studies indicate that astodrimer sodium nasal spray acts as a physical barrier between the highly positively charged SARS-CoV-2 spike protein and the negatively charged cellular proteins that act to concentrate virus near the angiotensin converting enzyme 2 (ACE2) receptor^13^.

SARS-CoV-2 receptors have been shown to be highly expressed in nasal epithelial cells^15^, such that intranasally administered therapeutic modalities could be effective in helping to prevent spread of infection of the virus. Astodrimer sodium has been formulated as a nasal spray, with the intention of being applied up to four times daily in the nasal cavity to help reduce exposure to infectious respiratory viral load. A nasal spray may be used in situations where there is increased risk of exposure, such as crowded places, public transport, or aged care/healthcare settings, before an infection with SARS-CoV-2 and other respiratory viruses develops. Like other marketed nasal sprays, astodrimer sodium nasal spray could also potentially be used after an infection to reduce viral load in the nasal cavity, and potentially reduce severity or duration of respiratory infection symptoms^16,17^.

Astodrimer sodium has previously been shown to be safe and well-tolerated in a vaginal gel also containing 1% astodrimer sodium that demonstrated efficacy for the treatment and prevention of bacterial vaginosis (BV)^18–20^. The size and negative charge of astodrimer sodium mean that it is not systemically absorbed following topical application to mucosal epithelia^21,22^.

Reports of transmission of SARS-CoV-2 by the fully vaccinated suggests an urgent need for additional therapeutic and prophylactic approaches against COVID-19^23^. Astodrimer sodium was reformulated into a nasal spray and assessed in a panel of nonclinical biocompatibility and toxicology studies. The product was shown to be not cytotoxic in vitro, was non-sensitizing in guinea pigs, was well tolerated and did not induce any signs of local or systemic toxicity, or systemic absorption of astodrimer sodium, when administered four times daily for 7 days in rats, and was well tolerated and did not induce any evidence of local irritation or other toxicity when administered four times daily for 14 days in rats.

The aim of current investigation was to assess the safety and tolerability of 1% astodrimer sodium nasal spray applied four times a day for 14 days in healthy volunteers. The frequency of product application of every 4 h was chosen based on data showing that iota-carrageenan, which is a negatively charged antiviral molecule that has similar chemical properties to astodrimer sodium, is retained on the nasal mucosa for approximately 4 h post administration^24^. The duration of product application of 14 days was chosen to simulate an intensive period of product application. The results of this investigation inform the likelihood of local and systemic side effects when the product is used to help protect against infection with, and potential transmission of, respiratory viruses.

## Methods

### Investigation design

**Figure 1:**
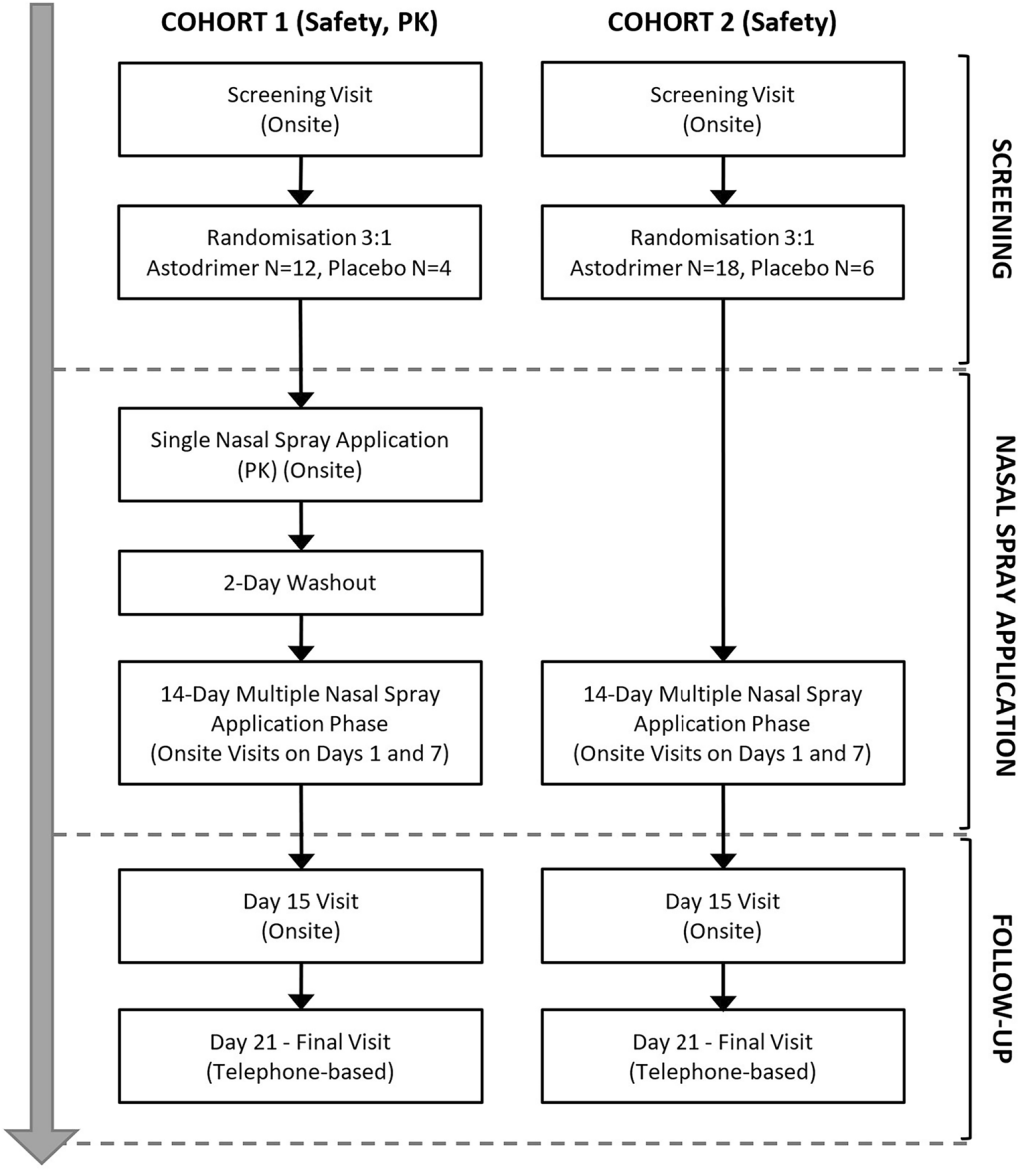
Investigation design. The investigation involved a screening phase, product application phase, and follow-up phase. Two cohorts of participants were enrolled, with participants in cohort 1 being assessed for safety and pharmacokinetics (PK, absorption), and participants in cohort 2 being assessed for safety.

This was a single-centre, double-blinded, randomized, placebo-controlled, exploratory clinical investigation of the safety, tolerability, and pharmacokinetics (PK), or absorption, of single and multiple applications of 1% astodrimer sodium nasal spray in healthy volunteers. An overview of the study design is presented in Fig. 1. The investigation was conducted at Linear Clinical Research, a dedicated clinical trials facility (WA, Australia). The investigation was approved by the Bellberry Human Research Ethics Committee (SA, Australia) on 21 December 2020. The study was conducted in accordance with all relevant guidelines and regulations including International Council on Harmonisation (ICH) Guidelines for Good Clinical Practice, The Declaration of Helsinki, and International Organization for Standardization (ISO) standard 14155:2020—Clinical investigation of medical devices for human subjects—Good clinical practice.

### Participants

All participants provided written informed consent prior to screening procedures. Eligible participants were males and nonpregnant, nonlactating females, aged 18 to 65 years, inclusive; who were in good general health with no current chronic diseases, normal physical examination and without clinically significant laboratory abnormalities; who had a body mass index (BMI) between 18 and 32 kg/m^2^ inclusive; and who did not smoke and had not used nicotine containing products within 2 months prior to screening. Females of child-bearing potential, or males sexually active with a female partner of child-bearing potential, had to be abstinent or agree to use effective contraceptive methods throughout the investigation.

Participants were excluded if they had clinically significant findings upon examination of the nasal cavity; had received another investigational medical device or investigational drug within 30 days or 5 half-lives of the drug (whichever was longer) prior to screening; had fever or a symptomatic viral or bacterial infection within 2 weeks of screening; had known allergy to astodrimer sodium or any of the spray components.

### Randomization and masking

The study enrolled 40 healthy volunteers into two cohorts. In Cohort 1, 16 participants were enrolled and randomized in a 3:1 ratio to astodrimer sodium nasal spray (N = 12) or placebo nasal spray (N = 4). In Cohort 2, 24 participants were enrolled and also randomized in a 3:1 ratio to astodrimer sodium nasal spray (N = 18) or placebo (N = 6). A computer generated (SAS 9.40 statistical software, SAS Institute, Inc., Cary, NC, USA) randomization list specifying a block size of 8 was used. A total of 5 blocks resulting in 40 randomization codes were generated. The randomization list was only accessible to the unblinded statistician and the pharmacist. Participants deemed eligible by the principal investigator were sequentially assigned randomization codes and dispensed astodrimer sodium nasal spray or placebo according to the randomization list by the pharmacist, who had no other involvement in the study.

The astodrimer sodium nasal spray and placebo nasal spray were packaged in identical containers with identical labelling. The placebo consisted of the same aqueous formulation as astodrimer sodium nasal spray, but without astodrimer sodium, and was identical in appearance and all other ways. The participants, investigator, and other site staff administering product or assessing outcomes were all masked to group assignment.

### Procedures

Astodrimer sodium nasal spray is a preserved (non-sterile) aqueous solution containing 1% (w/w) astodrimer sodium packaged in a container consisting of a bottle and a nasal pump (actuator) that delivers a nominal volume of 100 μL per actuation. The placebo nasal spray was identical and delivered the same volume. Both products are stored at room temperature.

Participants in Cohort 1 were administered a single pump actuation (spray) of study product to each nostril by appropriately trained investigational site staff to evaluate the safety, tolerability and absorption following single application. After a washout period ≥ 2 days, which was chosen based on clinical data showing that astodrimer sodium administered vaginally in a mucoadhesive gel formulation was detectable in small amounts in only some participants up to 24 h after dosing^25^, participants self-applied product four times a day at evenly spaced intervals of approximately 4 h and no less than 1 h, for 14 days, and then were off-treatment during a 7-day follow-up phase. The transition from a single application to four times daily application was deemed appropriate based on data from nonclinical studies of four times daily nasal application, as well as the existing body of nonclinical and clinical safety data for astodrimer sodium administered to other mucosal epithelia, showing an acceptable safety profile.

Participants in Cohort 2 followed the same schedule, excluding the single application phase. The application schedule resulted in a nominal daily exposure to 8 mg astodrimer sodium; and a total exposure of 112 mg over the complete 14-day application period.

Following a Screening visit, eligible participants attended on-site visits on Day 1 for commencement of product application (participants in Cohort 1 attended on-site for Day 1 of both the single and multiple application phases), Day 7 of the multiple application phase, and Day 15 for follow-up. A final visit was conducted by telephone at Day 21.

On Day 1 prior to product administration, all participants underwent a confirmatory eligibility assessment, a urine drug screen, an alcohol breath test, nasal cavity examination, and assessment for vital signs and blood chemistry. Female participants had a urine pregnancy test.

In addition to these assessments, on Day 1 of the single product application phase for Cohort 1, participants also had an ECG, and blood samples taken for analysis of astodrimer sodium levels prior to product application, and an ECG and vital signs assessed at 30 min, 1, 2, 3, and 4 h after, product application. Blood samples were taken for analysis of astodrimer sodium levels 15, 30 min, and 1, 2, 3, and 4 h after product application.

On Day 7, all of the respective assessments for each cohort, including blood samples for analysis of astodrimer sodium levels for Cohort 1, 15 min prior to and 1, 2, 3, and 4 h after one of the doses, but excluding urine pregnancy, drug screen and alcohol breath test, were repeated.

All participants underwent a nasal cavity examination, assessment for vital signs and blood chemistry, and an ECG at Day 15. Blood samples were not collected at Day 15 for analysis of astodrimer sodium levels due to the large body of existing nonclinical and clinical data showing lack of systemic absorption of astodrimer sodium following intensive topical application to mucosal epithelia, balanced with the aim to minimize burden of procedures for participants.

Blood samples for analysis of astodrimer sodium levels were collected in lithium heparin tubes and plasma was analyzed by micellar electrokinetic chromatography using a fully validated analytical method. If astodrimer sodium was detected in plasma, concentration values, concentration–time profiles and standard non-compartmental PK parameters were to be presented.

Incidence of treatment emergent adverse events (TEAEs) and concomitant medications were recorded at each on-site visit and at Day 21 via a telephone call follow-up.

At the times participants where not on-site, they were contacted by telephone to monitor TEAEs, concomitant medications and compliance with product application. Participants maintained a daily eDiary from Days 1 to 14, capturing information on product application, concomitant medications, and potential AEs. The eDiary was reviewed in between visits and at each on-site visit to assess product administration compliance and provide guidance as necessary, and to assess and record AEs and concomitant medications.

Safety oversight was provided by a safety monitoring committee (SMC) comprised of the Principal Investigator, Sponsor representatives, including medical expert, and independent Medical Monitor.

### Outcomes

The primary outcome measure of this investigation was the safety and tolerability of astodrimer sodium nasal spray, determined as the frequency and severity of TEAEs and serious adverse events (SAEs), including clinically significant nasal cavity examination findings, physical examination findings, vital signs, ECG and blood chemistry abnormalities. At each visit, the investigator determined whether any TEAEs had occurred by asking non-leading questions.

Compliance with required product use was determined by the number of applications reported and the reduction in weight of returned product bottles, and used as a measure of tolerability.

The secondary outcome measure of the investigation was the absorption of astodrimer sodium into blood following application to the nasal mucosa of healthy volunteers. This outcome was determined as plasma concentrations of astodrimer sodium after single or multiple applications at Day 7, and if detected, the derived PK parameters. Blood sampling was not repeated at Day 15 in order to limit burden on participants, as it was considered unlikely that absorption of astodrimer sodium would be detected based on prior evidence of lack of systemic absorption, and sampling at 7 days provided an assessment following intensive use of the product four times daily (24 to 28 applications).

### Statistical analysis

The sample size was not selected for inferential statistics but to allow sufficient qualitative evaluation of the safety of multiple nasal applications of astodrimer sodium nasal spray. It was assumed that the probability of observing at least one TEAE or SAE in a participant applying the product (i.e., event presence or absence) follows a binomial distribution. A sample size of 30 participants receiving astodrimer sodium nasal spray provided a high probability (> 95%) of observing at least one TEAE or SAE if the true event rate was greater than or equal to 1 in 10 users.

The primary safety analyses included all participants who were randomized and received any amount of astodrimer sodium nasal spray or placebo. The secondary assessment of absorption included all participants in Cohort 1 who were randomized and received any astodrimer sodium nasal spray.

Categorical endpoints were summarized by treatment group as frequencies, percentages of the total number of participants, and total number of events. TEAEs were coded using MedDRA v23.1. SAS 9.40 statistical software (SAS Institute, Inc., Cary, NC, USA) was used for all analyses.

### Ethics approval and consent to participate

The investigation was approved by the Bellberry Human Research Ethics Committee (EC00469; SA, Australia) on 21 December 2020. All participants provided written informed consent prior to screening procedures.

## Results

**Figure 2:**
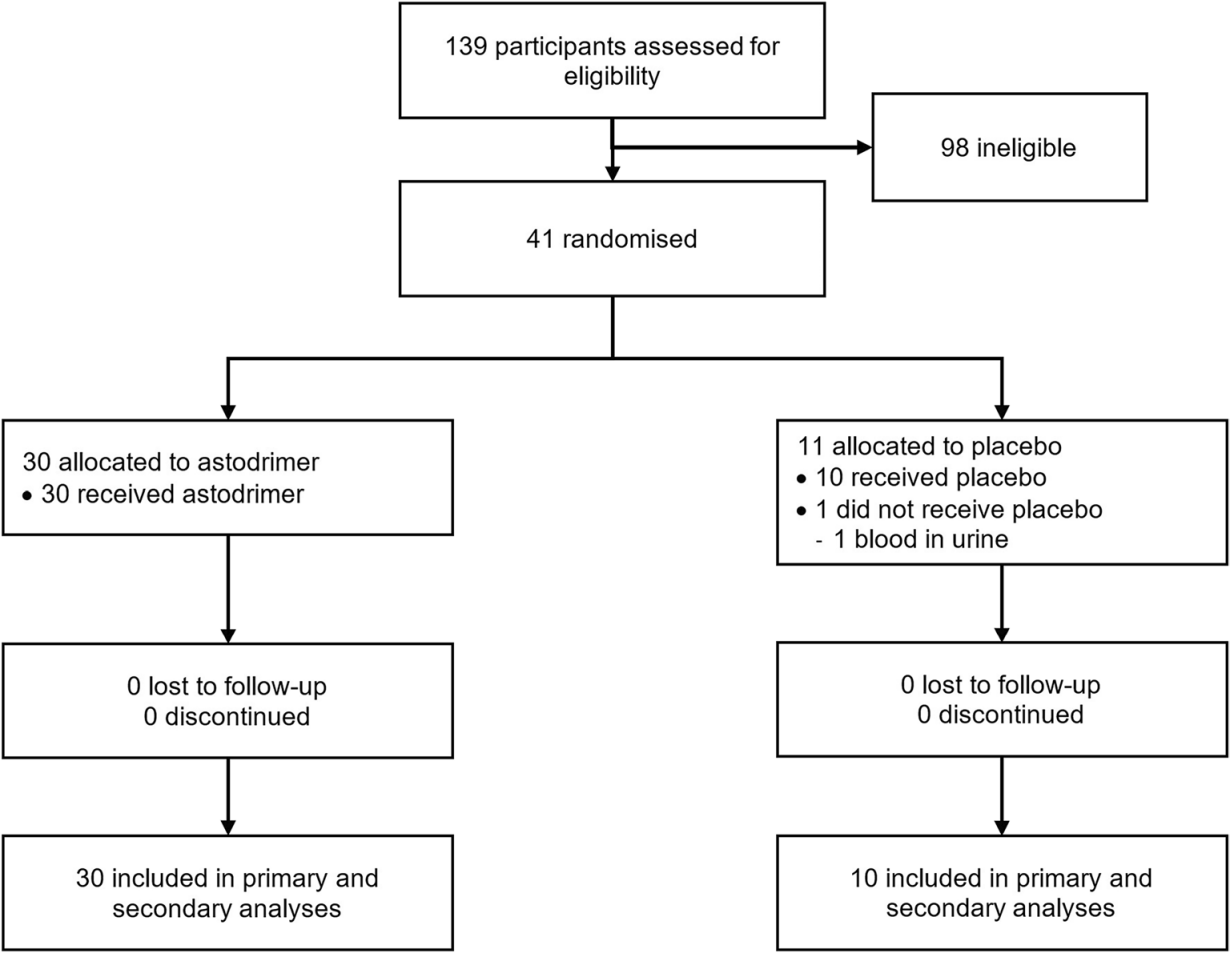
Trial profile.

The investigation was conducted from 6 January 2021 to 29 March 2021. There were 139 participants screened. Of these, 98 were excluded. All 40 participants who were randomized and received study product completed the investigation and were included in the primary analysis. All participants in Cohort 1 (N = 16) were included in the secondary analysis. A trial profile of participant disposition is shown in Fig. 2. There was one participant randomized to Cohort 2 (placebo) but excluded from the investigation due to blood on the urine dipstick at Day 1. This participant was replaced before they were administered study product.

Baseline demographics of the safety population are summarized in Table 1. Participants in astodrimer and placebo arms were of similar age. There was a higher proportion of female participants in the placebo arm (70.0% female) than in the astodrimer arm (50.0% female). BMI was similar between the groups.

**Table 1:** Baseline demographic characteristics of the safety population.

	Astodrimer nasal spray (N = 30)	Placebo nasal spray (N = 10)	Total (N = 40)
**Age, years**			
Mean (SD)	30.5 (9.7)	30.9 (9.9)	30.6 (9.6)
Range	19 to 58	20 to 47	19 to 58
**Sex, n (%)**			
Male	15 (50.0%)	3 (30.0%)	18 (45.0%)
Female	15 (50.0%)	7 (70.0%)	22 (55.0%)
**Ethnic origin, n (%)**			
Hispanic or Latino	2 (6.7%)	2 (20.0%)	4 (10.0%)
Not Hispanic or Latino	28 (93.3%)	8 (80.0%)	36 (90.0%)
**Race, n (%)**			
White	17 (56.7%)	6 (60.0%)	23 (57.5%)
Asian	7 (23.3%)	2 (20.0%)	9 (22.5%)
Black	1 (3.3%)	1 (10.0%)	2 (5.0%)
Other	5 (16.7%)	1 (10.0%)	6 (15.0%)
**BMI (kg/m**^**2**^**)**			
Mean (SD)	24.2 (2.8)	22.6 (3.6)	23.8 (3.1)
Range	19 to 31	18 to 31	18 to 31

*BMI* body-mass index, *SD* standard deviation.

For the primary analysis of safety and tolerability, a total of 47 TEAEs were recorded in 27 (67.5%) participants. Of these, 32 were recorded in 19 (63.3%) participants randomized to astodrimer, and 15 were recorded in 8 (80.0%) participants randomized to placebo (Table 2).

**Table 2:** Overall summary of treatment emergent adverse events.

	Astodrimer nasal spray (N = 30)		Placebo nasal spray (N = 10)		Total (N = 40)	
	Participants (%)	Events	Participants (%)	Events	Participants (%)	Events
Any TEAE	19 (63.3%)	32	8 (80.0%)	15	27 (67.5)	47
Any severe TEAE	0 (0%)	0	0 (0%)	0	0 (0%)	0
Any TEAE leading to discontinuation of study product	0 (0%)	0	0 (0%)	0	0 (0%)	0
Any serious TEAE	0 (0%)	0	0 (0%)	0	0 (0%)	0
**By relationship**						
Related	10 (33.3%)	15	5 (50.0%)	8	15 (37.5%)	23
Definitely related	3 (10.0%)	3	1 (10.0%)	1	4 (10.0%)	4
Probably related	1 (3.3%)	1	2 (20.0%)	4	3 (7.5%)	5
Possibly related	9 (30.0%)	11	3 (30.0%)	3	12 (30.0%)	14
Not related	13 (43.3%)	17	5 (50.0%)	7	18 (45.0%)	24
Probably not related	8 (26.7%)	10	1 (10.0%)	1	9 (22.5%)	11
Not related	6 (20.0%)	7	5 (50.0%)	6	11 (27.5%)	13
**By severity**						
Grade 1: mild	19 (63.3%)	32	8 (80.0%)	15	27 (67.5)	47
Grade 2: moderate	0 (0%)	0	0 (0%)	0	0 (0%)	0
Grade 3: severe	0 (0%)	0	0 (0%)	0	0 (0%)	0
Grade 4: life threatening	0 (0%)	0	0 (0%)	0	0 (0%)	0

*TEAE* treatment emergent adverse event.

There were no SAEs, nor TEAEs leading to withdrawal from use of study product. All TEAEs were of mild severity and all were self-limiting, with the exception of some events such as headache, and musculoskeletal aches and pains, for which common pain medications were used.

Of the 47 TEAEs, 23 were deemed by the investigator to be potentially related to the study product (possibly related, probably related or definitely related) and 24 were deemed to be unrelated (not related or probably not related) (Table 2). Of the 23 TEAEs deemed potentially related, 15 were recorded in 10 (33.3%) participants randomized to astodrimer, and 8 were recorded in 5 (50.0%) patients randomized to placebo.

Overall, the most commonly experienced TEAE was headache, with 9 participants (30.0%) in the astodrimer arm and 2 participants (20.0%) in the placebo group reporting headache. Other TEAEs that were experienced by at least 5% more participants in the astodrimer arm than the placebo arm were tension headache (N = 2; 6.7% vs 0), nausea (N = 2; 6.7% vs 0) and rhinorrhoea (N = 2; 6.7% vs 0). Overall, upper respiratory tract TEAEs were experienced by a greater proportion of participants in the placebo arm (N = 5; 50.0%) than in the astodrimer arm (N = 9; 30.0%).

Of the 23 potentially related TEAEs (Table 3), 4 were deemed “definitely related” and were cases of nasal discomfort during product application, described as a transient tingling sensation, that occurred at the same frequency in each arm (astodrimer N = 3, 10.0%; placebo N = 1, 10.0%).

**Table 3:** Incidence of treatment emergent adverse events considered potentially related.

	Astodrimer nasal spray (N = 30)		Placebo nasal spray (N = 10)		Total (N = 40)	
	Participants (%)	Events	Participants (%)	Events	Participants (%)	Events
Headache	4 (13.3%)	5	1 (10.0%)	1	5 (12.5%)	6
Epistaxis	3 (10.0%)	3	1 (10.0%)	1	4 (10.0%)	4
Nasal discomfort	3 (10.0%)	3	1 (10.0%)	1	4 (10.0%)	4
Nasal congestion	2 (6.7%)	2	2 (20.0%)	2	4 (10.0%)	4
Nausea	1 (3.3%)	1	0 (0.0%)	0	1 (2.5%)	1
Rhinorrhoea	1 (3.3%)	1	0 (0.0%)	0	1 (2.5%)	1
Nasal pruritus	0 (0.0%)	0	1 (10.0%)	1	1 (2.5%)	1
Paranasal sinus discomfort	0 (0.0%)	0	1 (10.0%)	1	1 (2.5%)	1
Throat irritation	0 (0.0%)	0	1 (10.0%)	1	1 (2.5%)	1

A further 5 TEAEs were deemed “probably related” and included nasal congestion (astodrimer N = 1, 3.3% v placebo N = 1, 10.0%), headache (placebo N = 1, 10.0%), paranasal sinus discomfort (placebo N = 1, 10.0%), and nasal pruritus (placebo N = 1, 10.0%); and 14 were deemed “possibly related” and included nasal congestion (astodrimer N = 1, 3.3%; placebo N = 1, 10.0%), headache (astodrimer, N = 4, 13.3%), epistaxis (astodrimer N = 3, 10.0%; placebo N = 1, 10.0%), nausea (astodrimer, N = 1, 3.3%), throat irritation (astodrimer, N = 1, 3.3%) and rhinorrhoea (placebo N = 1, 10.0%). There were no TEAEs considered potentially related to study product that were experienced by 5% or more participants in the astodrimer arm than the placebo arm.

No clinically significant findings were recorded during the nasal cavity examinations, and no clinically relevant laboratory abnormalities, vital signs or ECG alterations, or physical examination findings were recorded during the investigation.

Compliance with required study product use, as determined by number of applications, was extremely high (mean 99.9% for astodrimer and 100% for placebo). Reduction in weight of the returned (and used) spray containers, showed that 76.7% (23/30) and 90.0% (9/10) for astodrimer and placebo, respectively, used the expected amount of nasal spray assuming a spray volume of 100 µL. Based on the recorded number of applications, total astodrimer exposure over the 14-day multiple application period averaged 106.4 mg, suggesting a mean daily exposure of 7.6 mg (95% of the nominal daily dose). The minimum exposure was 71 mg and the maximum exposure was 147 mg, resulting in mean daily exposures of 5.1 mg (64% of the nominal daily dose) and 10.5 mg (131%), respectively.

For the secondary analysis, astodrimer sodium was not detected in any of the collected plasma samples (lower limit of quantitation: 0.75 µg/mL [45 nM]). Therefore, no derived PK parameters could be calculated.

## Discussion

Nasal application of 1% astodrimer sodium nasal spray was well tolerated when used four times a day for 14 days in healthy volunteers. TEAEs occurred in a greater proportion of participants in the placebo arm than the astodrimer arm, but the difference is not considered clinically significant, and all were of mild intensity and mainly self-limiting. TEAEs considered potentially related to study product also occurred in a greater proportion of participants receiving placebo than astodrimer sodium, but again, the difference is not considered clinically significant. Nasal discomfort during product application, described as a transient tingling sensation lasting a few seconds, occurred at the same frequency in each arm, suggesting this sensation is related to the physical administration of the formulation and not due to the presence of astodrimer sodium. No participants experienced serious AEs, or TEAEs leading to withdrawal from the study. No systemic absorption of astodrimer sodium was detected, indicating the risk of systemic adverse effects is negligible.

This was the first investigation of the safety of a nasal spray formulation containing 1% w/w astodrimer sodium applied to the nasal mucosa of humans. However, the safety, tolerability and efficacy of astodrimer sodium in a vaginal gel formulation, which has a near identical formulation to the nasal spray, with the exception of the amount and type of carbomer used, has been evaluated in multiple phase 1, 2 and 3 clinical trials for the treatment and prevention of BV.

These previous clinical investigations of astodrimer gel showed that astodrimer sodium is safe and well tolerated when applied vaginally every day for 7 days and every other day for up to 16 weeks, and is not systemically absorbed^18–20^. A recent meta-analysis of these phase 2 and 3 clinical studies confirmed that astodrimer gel was superior to placebo in treating patients with BV^26^. When compared to placebo, the meta-analysis showed that the incidence of severe AEs was significantly lower in the astodrimer gel group. Astodrimer gel also demonstrated equivalent tolerability to placebo, except for the incidence of vulvovaginal candidiasis, which was higher for astodrimer. However, the rate of candidiasis for astodrimer gel compares favorably with other products used for treatment of women with BV. Therefore, the findings of a benign safety and tolerability profile of the nasal spray formulation in the current investigation are consistent with the findings from previous clinical studies of astodrimer gel.

Astodrimer sodium has antiviral and virucidal activity against a broad spectrum of respiratory viruses, including SARS-CoV-2 in vitro^12,13^ and in vivo^14^, SARS-CoV-1 and MERS-CoV in vitro^13^, and common cold human CoV (229E, OC43, and NL63), human respiratory syncytial virus, human rhinovirus and human influenza A viruses in vitro.

Astodrimer sodium retains activity against SARS-CoV-2 in the presence of a nasal mucosal simulant^12^ and has been shown to possess activity against all identified variants of concern of SARS-CoV-2^13^. Astodrimer sodium has also been shown to retain antiviral activity ex vivo following vaginal administration in humans^25^.

Astodrimer sodium mimics negatively charged heparan sulfate, which is used by viruses to concentrate at a cell surface, and provides a potent antiviral and virucidal barrier to viral attachment and entry into a host cell. Astodrimer sodium forms multiple electrostatic interactions with the spike or binding proteins on viruses that are not negated by differences in the binding domains of different types or strains of virus^13^.

Therefore, astodrimer sodium nasal spray is intended to be used for protection against broad-spectrum respiratory virus infection.

The current evidence supports that the predominant route of SARS-CoV-2 infection is by direct contact with respiratory droplets^27^. Current COVID-19 vaccines are effective at preventing severe disease but do not completely prevent viral infection. The recent reports of the SARS-CoV-2 Delta and Omicron variants infecting vaccinated, partially vaccinated and unvaccinated people illustrates the SARS-CoV-2 infection remains an imminent threat to the world’s most vulnerable people. A nasal spray to help protect the nasal mucosa from pathogenic respiratory viruses would be a valuable tool to use in concert with vaccines and other public health measures^23^. While efficacy studies in humans are yet to be conducted for astodrimer sodium nasal spray, astodrimer sodium has shown favorable antiviral properties compared with iota-carrageenan^12^, which has itself been shown to reduce viral load and positively impact symptoms of respiratory infections when administered as a nasal spray^16,17^.

There were no clinically significant findings from the nasal cavity examinations, remarkable laboratory abnormalities, vital signs or ECG alterations or physical examination findings during the investigation of astodrimer nasal spray. Participants were compliant with product application as measured by number of applications and the weight of the returned study product. The discrepancy between number of applications and weight reduction for a small number of participants, and the range of minimum and maximum amounts of astodrimer administered, suggest there may be variability in the amount of nasal spray ejected from the pump by users. As astodrimer sodium is applied and acts topically in the nasal cavity and is not required to be systemically absorbed or delivered in precise amounts in order to achieve its action, some variability in spray administration within or between users is not expected to impact the performance of astodrimer sodium nasal spray at blocking respiratory viruses.

The finding that astodrimer sodium was not detected in the bloodstream following repeated nasal application is consistent with previous extensive nonclinical and clinical data showing lack of absorption into the bloodstream following topical application to mucosal membranes^21,22^. Therefore, systemic adverse events would not be expected to occur from nasal administration of astodrimer sodium, and there was no evidence of such events occurring in the current investigation.

The sample size in the current investigation provided a high probability of detecting very common adverse events in people using astodrimer nasal spray. Given the lack of systemic absorption, less common, or rare, side effects are not expected with nasal administration of the product. Additional data on less common side effects could be investigated in larger clinical investigations or from post-market surveillance data.

## Conclusions

In conclusion, astodrimer sodium 1% nasal spray was well tolerated in healthy adult volunteers, with no systemic absorption of astodrimer sodium being detected. TEAEs occurred in a greater proportion of participants receiving placebo than astodrimer but this difference is not considered clinically significant and all TEAEs were of mild intensity. The safety profile of the mucoadhesive barrier nasal spray with 1% astodrimer sodium, combined with its broad-spectrum antiviral and virucidal activity, mean that the product is well suited to topical nasal administration to help protect a user from acute respiratory virus infection, or reduce viral exposure to a level that would be easily detected and destroyed by the innate immune system prior to systemic infection or potentially cell-damaging proinflammatory responses. Astodrimer sodium nasal spray is a promising tool warranting further investigation and extension of the clinical data for its potential to combat current and future endemic, epidemic and pandemic emergent respiratory viral pathogens.

## Data Availability

The datasets used and analyzed during the current study are available from the corresponding author on reasonable request after approval of a proposal and under a signed data access agreement.
